# Large mobile thrombus in non-atherosclerotic thoracic aorta as the source of peripheral arterial embolism

**DOI:** 10.1186/1477-9560-3-19

**Published:** 2005-11-29

**Authors:** Nasser M Malyar, Rolf A Janosi, Zoran Brkovic, Raimund Erbel

**Affiliations:** 1 Department of Cardiology, West German Heart Centre, University, Duisburg-Essen, Germany; 2 Department of Angiology, University, Duisburg-Essen, Germany

## Abstract

The presence of thrombi in the atherosclerotic and/or aneurysmatic aorta with peripheral arterial embolism is a common scenario. Thrombus formation in a morphologically normal aorta, however, is a rare event. A 50 years old woman was admitted to the mergency department for pain, coldness, and anesthesia in the the left foot. She had a 25 years history of cigarette smoking, a history of postmenopausal hormone replacement therapy (HRT), hypercholesterolemia and hyperfibrinogenemia. An extensive serologic survey for hypercoagulability, including antiphospholipid antibodies, and vasculitis disorders was negative. Transesophageal echocardiography revealed a large, pedunculated and hypermobile thrombus attached to the aortic wall 5 cm distal of the left subclavian artery. The patient was admitted to the surgery department, where a 15 cm long fresh, parietal thrombus could be removed from the aorta showing no macroscopic wall lesions or any other morphologic abnormalities.

This case report demonstrates the possibility of evolving a large, pedunculated thrombus in a morphologically intact aorta in a postmenopausal woman with thrombogenic conditions such as hyperfibrinogenemia, hypercholesterolemia, smoking and HRT. For these patients, profiling the individual risk and weighing the benefits against the potential risks is warranted before prescribing HRT.

## Background

The cardiac cavities are in >85 % of cases the primary source of peripheral arterial embolism (PAE). Due to new and sophisticated imaging techniques in recent years, such as spiral computed tomography (CT) scan, magnetic resonance imaging (MRI) and transesophageal echography (TEE), noncardiac sources of PAE has been detected with increasing frequency. Among these non-cardiac sources, the aorta has been reported in up to 5 % of cases to be the origin of PAE [1]. However, while mural thrombus in the aneurysmatic or atherosclerotic aorta with protruding atheromas may be the source of major arterial embolism [2], emboli originating from a non-atheromatous and non-aneurysmatic aorta is a rare event [3]. We report a case of pedunculated, large and highly mobile thrombus, formed within a morphologically normal descending thoracic aorta, resulting in recurrent peripheral embolism that caused acute ischemia of the lower extremities.

## Case report, diagnostic workup and therapeutic intervention

**Figure 1:**
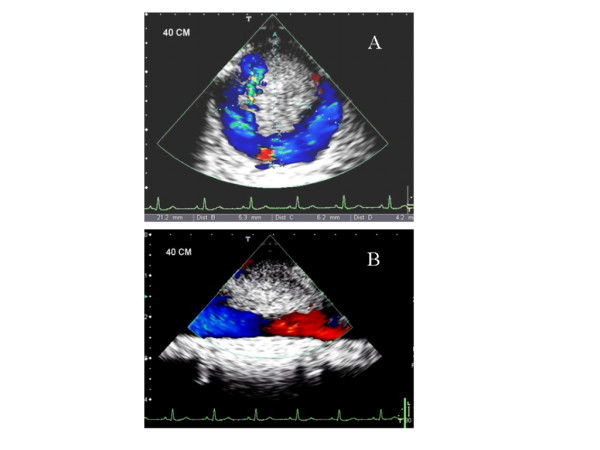
Transesophageal echocardiographic cross-sectional (Panl A) and longitudinal (Panel B) images of the descending thoracic aorta showing the highly mobile, floating thrombus. The original lumen of the aorta (21 mm) is reduced by the thrombus to a circumferential patent lumen of 4–6 mm.

**Figure 2:**
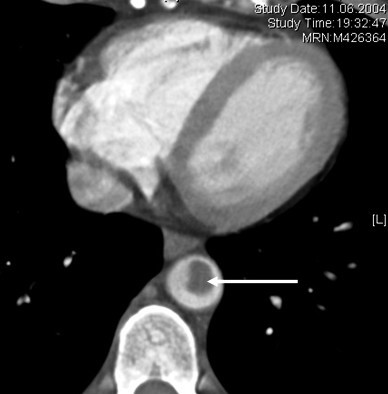
Thoraco-abdominal CT-scan image showing the thrombus in the thoracic aorta (arrow).

A 50 years old woman was admitted to the emergency department because of acute pain, coldness, and anesthesia in the left foot. Pulses at the left popliteal, tibialis posterior and at the dorsalis pedis artery were not palpable. For cardiovascular and prothrombotic risk factors, she had a 25 years history of cigarette smoking (30–35 cigarettes/day), a history of 16 months of postmenopausal hormone replacement therapy (HRT) by oral intake of an estrogen-gestagen combination (Presomen, 0.6 mg/d for 16 months) and untreated hypercholesterolemia (5.90 mmol/L, reference: <5.2 mmol/L). She had no previous medical history of arrhythmia, ischemic heart disease, diabetes mellitus, or stroke. Duplex sonographic examination of the arterial system of the limbs revealed the acute thromboembolic occlusion of the left popliteal artery. Diagnostic work up was initiated to determine the source of the embolism. The resting ECG and a holter monitoring for 24 h disclosed normal sinus rhythm without any pathologic findings. The transthoracic echocardiographic examination (TTE) showed no structural or functional cardiac abnormalities. Subsequently, a TEE was performed for evaluation of the cardiac cavities and of the thoracic aorta, revealing a large, pedunculated and hypermobile thrombus (Figure 1, Panl A and B) attached to a non-atherosclerotic aortic wall caudal of the left subclavian artery. CT scans of the thoraco-abdominal aorta (Figure 2) confirmed the presence of the thrombus seen in TEE. The entire thoracic aorta had normal dimensions with no visible signs of atherosclerosis. For biochemical laboratory parameters, thrombocytes (530*10^9^/L, reference: <410*10^9^/L), fibrinogen (5.5 g/L, normal: <3.50 g/L) and total Cholesterol (5.90 mmol/L, reference: <5.2 mmol/L) were elevated. All other parameters including glucose (5.8 mmol/L) and homocysteine (8.6 μmol/L) levels were within the reference limit. An extensive serologic survey for hypercoagulability, including antiphospholipid antibodies, and vasculitis disorders was negative.

Because of the high risk for recurrent peripheral embolization due to the size and the hypermobility of the thrombus, the patient was admitted to the vascular surgery unit, where a 15 cm long thrombus in the thoracic aorta with its origin 5 cm distal of the left subclavian artery could be removed. During surgery no macroscopical signs of intimal lesions of the aorta could be detected. Histological evaluation of the thrombus revealed a red, parietal thrombus without any evidence of malignancy. The postoperative course was uneventfull and the patient was discharged 10 days after the surgical intervention. The patient was treated with vitamin-k-antagonist phenprocoumon (INR: 2.5–3.5) for secondary prevention of thromboembolism and with 10 mg/d of atorvastatin for hypercholesterolemia additional to cessation of smoking and oral intake of HRT. On this therapeutic regimen, the patient was asymptomatic with no pathologic findings in the imaging workup at six months follow up.

## Discussion

In this case report we demonstrate the presence of a large, mobile thrombus in a morphologically normal aorta as the source of PAE. While thrombi in aneurysmatic or atherosclerotic aorta, especially in combination with pro-thrombotic risk factors such as hypercoagulability, antiphospholipid syndrome, disorders of protein C and S and vasculitis, have previously been described, the presence of thrombi in a non-atherosclerotic aorta is a rare event. As the aorta of the patient described in this case report had normal dimensions and was free of any visible atherosclerotic intimal lesions, the additive prothrombotic effects of hypercoagulability, due to increased level of fibrinogen, thrombocytes and cholesterol, and of oral HRT and excessive smoking seem most likely to be the cause of the thrombus formation.

Plasma fibrinogen is an important component of the coagulation cascade, as well as a major determinant of blood viscosity and blood flow. Increasing evidence from epidemiological studies suggests that elevated plasma fibrinogen levels are associated with an increased risk of cardiovascular disorders, including ischemic heart disease, stroke and thromboembolism [4]. The increase in plasma fibrinogen levels may promote a prothrombotic or hypercoagulable state, and may in part explain the risk of stroke and thromboembolism. The independent and close relation between fibrinogen and cardiovascular risk including thromboembolic complications in the peripheral arterial system has been documented in a substantial number of studies. Fibrinogen strongly affects blood coagulation, blood rheology and platelet aggregation. In addition, it has direct effects on the vascular wall and is a prominent acute phase reactant [5,6].

The adverse cardiovascular effects of HRT have been well documented in the Women's Health Initiative (WHI) trial [7-11]. The combined estrogens-progestin arm of the WHI trial [8] demonstrated that some risks, such as for thromboembolism, coronary heart disease, and stroke, arise within the first 1 to 2 years of therapy, while other risks such as the risk for breast cancer appear to increase with longer-term hormone therapy. In our patient, the thromboembolic event occurred 16 months after the initiation of the HRT, which is consistent with the observation in the WHI study. The recently published clinical recommendations and guidelines[12] by the US Preventive Services Task Force (USPSTF) on hormone therapy for the prevention of chronic conditions in postmenopausal women consider the harmful effects of combined estrogens and progestin likely to exceed the benefits of chronic disease prevention. Therefore, it is recommended that the decision of HRT for postmenopausal complaints should be based on weighing the possible harm arising from the individual risk factors and the benefit.

The combination of smoking and HRT is a substantial risk factor for cardiovascular complications and a burden for the public health care, regrading the fact that products for HRT in the postmenopause are the second most frequently prescribed drugs in the USA [13,14] and 50 % of women on HRT are smokers [8]. Smoking per se not only increaes the incidence of thrombotic events, it has also been shown to decrease the level of serum estrogens by 50 % [15], thereby reducing, or even completely abolishing, the well-established beneficial effects of estrogen such as reducing hot flushes, prevention of osteoporosis and the positive effect on lipid metabolism [13,14]. Cigarette smoking induces global changes in both peripheral and central vascular function [16]. Some authors postulate that the most important effects of cigarette smoking in promoting atherosclerosis and thromboembolic complications may be endothelial disturbance and fibrin formation [17]. Consequently, cessation of smoking is a crucial preventive step towards minimizing the thromboembolic risks.

Generally, thromboembolic events are associated with advanced age, complex and ulcerated atherosclerotic plaques to which the thrombus is attached. Interestingly, the region around the left subclavian artery seem to be one of the predisposed localization for thrombus formation [18]. However, as this case report in accordance with few previously published studies demonstrate, it can also affect younger patients. In the study by Laperche et al. [18] the mean age of the 23 patients with aortic thrombus was 45 ± 8.4 years, with the youngest patient being 34 years old. In their study, smoking was the leading risk factor (n = 16) followed by hypercholesterolemia (n = 11) and elevated fibrinogen levels (n = 10). In our patient all 3 risk factors were present. In the study by Laperche et al. [18] histopathological examination revealed microscopic features of atherosclerosis limited to the insertion site in all patients undergoing surgery for thrombectomy. Considering the high thrombogenic potential of atherosclerotic plaques [19], it is likely that the thrombus in our patient was attached to an atherosclerotic plaque, even when it was not visible on TEE. Perler et al. [20] reported previously about thromboembolic events originating from a morphologically normal aorta in two young women. Both patients were taking oral steroidal medications, and both patients were heavy cigarette smokers. These cases and a review of the previous literature suggest that the development of aortic mural thrombi, at least in some patients, may not always result from diffuse and advanced atheromas, but may constitute a separate and distinct clinical entity of a premature atherosclerosis.

Detection of mobile thrombus of the thoracic aorta has become increasingly common thanks to routine exploration using TEE after any embolic events. TEE has been shown to detect reliably aortic thrombi [21]. Using high-frequency, multiplane probes, TEE sufficiently allows not only the detection, it also provides such important information as the size, the location and the implantation base of the thrombus. It has previously been demonstrated that these information obtained from TEE correlate well with information obtained from macroscopic examination [18]. The high spatial resolution allows detailed examination of the entire thoracic aorta as well as the intimal surfaces. Additionally, the real-time imaging provides information such as the mobility of the thrombus, which is not possible with CT. Such additional information, however, is helpful for immediate and long-term therapeutic decision making. However, it must be emphasized that the resolution capability of TEE is not sufficient to detect accurately atherosclerotic changes in the aortic wall at the microscopic level, as is possible with intravascular ultrasound.

The abdominal aorta also constitutes a frequent origin for thromboembolism [1,22]. This region, however, is not accessible to TEE. Therefore, optimal approach for a complete diagnostic workup for search of the thromboembolic sources requires the synergistic use of at least two modalities, i.e. TEE (for exploration of the cardiac and thoracic aorta origins) and CT or MRI (for exploration of the abdominal origins). The ubiquitaer availability, the excellent sensitivity and specificity and immediate results make TEE as the preferred and primarily used imaging modality for diagnostic evaluation in such cases.

Several treatments have been used in different forms with variable success for management of aortic thrombus, including anticoagulant therapy alone [18,23], thrombolysis [24], thromboaspiration and surgery [25]. At our institution, we initiate immediate anticoagulant therapy with heparin for 2 weeks after the thrombus is specified. At the end of 2 weeks TEE (alternatively MRI) is performed. If the thrombus shows reduction in size, the heparin therapy is continued until the thrombus resolves. If anticoagulant therapy fails, surgical intervention is considered. Surgical intervention is preferred to anticoagulant regimen in young patients, in the presence of a large hypermobile thrombus (as in this case report) and in patients with recurrent embolic events. Similar therapeutic strategies have also been suggested by others [18,25,26]. Endovascular stent-grafting provides a new minimally invasive therapeutic option in the treatment of symptomatic mobile thoracic aortic thrombus [27], however, its role in the setting of aortic thrombosis regarding the long term outcome is not established yet.

Evidence-based recommendations and guidelines regarding the therapeutic management and the long-term surveillance are lacking. Prospective studies are required to address the issue of evidence based guidelines and optimal approaches for the diagnostic, therapeutic and follow up management of these patients.

## Conclusion

Embolic events arising from thrombi within a non-aneurysmatic, non-atherosclerotic aorta is a rare but possible emergency event. Physicians have to be aware of the higher risk for developing thromboembolic complication in women under HRT in presence of additional prothrombotic risk factors such as smoking and hypercoagulability, not only in the venous but also in the arterial vascular system; therefore, profiling the individual risk and weighing the benefits against the potential risks is warranted before prescribing HRT. Transesophageal echocardiography serves as the most appropriate initial imaging modality for diagnostic work up and therapeutic management in patients with peripheral embolic events.

## List of abbreviations

CT: computed tomography

HRT: hormone replacement therapy

MRI: magnetic resonance imaging

PAE: peripheral arterial embolism

TTE: transthoracic echocardiography

TEE: transesophageal echocardiography

## Competing interests

The author(s) declare that they have no competing interests.

## Authors' contributions

N.M.M.: tacked out the diagnostic workup of the patient, carried out the TTE and TEE examinations and drafted the manuscript; R.A.J: tacked the MRI and CT examinations and contributed to the writing of the manuscript; Z.B.: carried out the therapy evolution of the patient; R.E.: revised the article critically for important intellectual content and have given final approval of the version to be published. All authors read and approved the final manuscript.
